# Male Weasels Decrease Activity and Energy Expenditure in Response to High Ambient Temperatures

**DOI:** 10.1371/journal.pone.0072646

**Published:** 2013-08-16

**Authors:** Karol Zub, Quinn E. Fletcher, Paulina A. Szafrańska, Marek Konarzewski

**Affiliations:** 1 Mammal Research Institute PAS, Białowieża, Poland; 2 Institute of Biological and Environmental Sciences, University of Aberdeen, Aberdeen, United Kingdom; 3 Institute of Biology, University of Białystok, Białystok, Poland; The University of Wollongong, Australia

## Abstract

The heat dissipation limit (HDL) hypothesis suggests that the capacity of endotherms to dissipate body heat may impose constraints on their energy expenditure. Specifically, this hypothesis predicts that endotherms should avoid the detrimental consequences of hyperthermia by lowering their energy expenditure and reducing their activity in response to high ambient temperatures (T_a_). We used an extensive data set on the daily energy expenditure (DEE, n = 27) and the daily activity time (AT, n = 48) of male weasels (*Mustela nivalis*) during the spring and summer breeding season to test these predictions. We found that T_a_ was related in a “hump-shaped” (i.e. convex) manner to AT, DEE, resting metabolic rate (RMR) and metabolic scope (the ratio of DEE to RMR). These results support the HDL hypothesis because in response to warm T_a_s male weasels reduced their AT, DEE, and RMR. Although the activity and energy expenditure of large endotherms are most likely to be constrained in response to warm T_as_ because they are less able to dissipate heat, our results suggest that small endotherms may also experience constraints consistent with the HDL hypothesis.

## Introduction

The thermal neutral zone of birds and mammals describes the range of ambient temperatures (T_a_) over which they do not have to expend additional energy to maintain their body temperature [1]. Although, the majority of research has focused on the energetic consequences of cold T_a_s, high temperatures are also energetically and physiologically challenging for animals. For example, studies on racing horses and dogs, as well as on exercising humans, have demonstrated that challenges associated with dissipating heat in response to hot and humid conditions not only reduces locomotor performance, but may also cause animals to die as a result of hyperthermia [2], [3].

The recently formulated heat dissipation limit (HDL) hypothesis proposes that maximal energy expenditure is not constrained by the availability of energy in the environment, but instead by the capacity of animals to dissipate body heat [4]. Originally, the HDL hypothesis was formulated to explain the ability of lactating laboratory mice to increase milk energy output when their capacity to dissipate body was experimentally increased [5], [6]. Excessive heat production during lactation is attributed to the high energetic costs of energy processing and milk production. However, the HDL hypothesis may also be relevant for explaining patterns in other life-stages (e.g. migration [7]), in addition to explaining broader ecological (e.g. Bergmann's rule [4]), evolutionary (e.g. senescence [8]), and inter-specific patterns (e.g. avian plumage differences [9]).

Tests of the HDL hypothesis in free-ranging endotherms (i.e. birds and mammals) critically require simultaneous estimates of energy expenditure, activity patterns, and T_a_, which are rarely obtained in the wild [10]–[13]. The HDL hypothesis predicts that in response to warm T_a_s that endotherms decrease both their daily activity time (AT) and daily energy expenditure (DEE) because they are unable to sufficiently dissipate heat to avoid the suite of negative effects that are associated with hyperthermia (reviewed in [4]). This hypothesis assumes that free-ranging endotherms, especially during the breeding period, are operating at a limit of energy expenditure [14]–[16], and that endotherms experience fitness-benefits as a result of being active, and thus expending energy (e.g. increased mating opportunities [17]). An alternative hypothesis suggests that in response to the T_a_s that animals experience in the wild, individuals can sufficiently dissipate the heat required to avoid hyperthermia. As a result, this alternative hypothesis would predict that mammals would not decrease their activity time (AT) and daily energy expenditure (DEE) in response to warm T_a_s.

Here, we used extensive data on the AT and DEE of male least weasels (*Mustela nivalis,* henceforth weasels) during the breeding season to test the predictions of the HDL hypothesis. Weasels are primarily diurnal predators [18], characterized by a short expected life span (< 1 year [19]). The AT and DEE of male weasels may be limited by their ability to dissipate heat during the breeding season because they engage in intensive mate searching throughout the breeding season [19]. In mammals with similar mating systems, mate searching has been shown to be energetically costly and associated with fitness-benefits [16], [17]. As a result, we assumed that weasels should aim to maximize their AT and DEE when actively searching for mates or hunting. If weasels decreased their AT and DEE in response to warm T_a_s, we interpreted this as support for the HDL hypothesis. Conversely, there was also good reason to believe that patterns of AT and DEE in male weasels during the breeding season would not support the HDL hypothesis. Specifically, the small size and elongated body shape of weasels increases their capacity to dissipate heat, and thus, makes them less prone to hyperthermia than animals of similar body mass, but with less elongated body shapes.

In addition to quantifying the DEE and AT of individual weasels, we also quantified the resting metabolic rate (RMR) of the same individuals during the breeding season. RMR quantifies the energetic demands required to meet only the basic physiological processes of an endotherm in thermoneutral conditions (see [1] for a review). RMR differs slightly from basal metabolic rate (BMR), which additionally requires that animals are non-growing, non-reproductive, and post-absorptive [1]. Quantifying RMR allowed us to calculate the metabolic scope (DEE/RMR) of individual weasels, which is the most common measure of how vigorously free-ranging animals expend energy over multiple-day periods [20].

## Materials and Methods

### Ethics statement

Weasels were captured and handled in strict accordance with the guidelines set by the Polish Committee on the Ethics of Animal Experiments. The protocol was approved by the Local Committee on the Ethics of Animal Experiments at the Medical University in Białystok (permits no. LKE 2003/04 and LKE 2004/06). Weasels are protected in Poland and were trapped under the auspices of Polish nature conservancy authorities (permits no. DOPweg- 4201-04-6/03/jr and DOPog-4201-04-43/05/aj). Weasels were fitted with neck-collar radio transmitters under ketamine-xylazine anesthesia and blood sampling was done under halothane anesthesia, to minimize suffering.

### Study area and animals handling

We studied a natural population of weasels in the Białowieża Forest (52.70^o^N, 23.86^o^E) of NE Poland. The climate in this area is transitional between temperate and continental, with average daily temperatures of 19.2°C in July and –3.2°C in January [21]. We trapped weasels in two types of habitats (meadows and river valleys), with 2 replicates within each habitat (4 trapping sites in total), representing typical habitats used by weasels during the day to hunt [22]. Animals were captured as described by [23]. Briefly, we used 20–40 traps at each trapping site arranged in transects that followed fences, ditches, roads and other linear features of the landscape, which are preferred by weasels [23]. T_a_ data were recorded at a meteorological station located within 1 km of the study sites. We used the temperatures recorded at 08:00, 14:00, and 20:00 to calculate the mean daily T_a_. Mean daily T_a_ correlated strongly with maximum daily T_a_ (R = 0.93), but was on average 5.2°C (SD = 1.7°C) lower than maximum daily T_a_. All data were collected from 22-Apr to 25-Oct between 2004 and 2007.

In total, 48 adult males were fitted with radio-collars to facilitate positional tracking of animals every 15 min during the daylight hours, over two-three consecutive day periods. The radio-tracking data were then used to calculate AT (h/day). If an animal was active when bearings were taken (i.e. the position of the signal changed in direction and intensity) we assumed it was active over the entire 15 min period. The majority of the radio-collared individuals (n = 27) were simultaneously injected with doubly labelled water (DLW) to measure their DEE [24], [25].

### Daily energy expenditure

DEE (kJ/day) was quantified 27 times in 25 individuals (two DEE values from two individuals) in 2004 (n = 5), 2005 (n = 8), 2006 (n = 11), and 2007 (n = 3). Briefly, DLW, with known quantities of the isotopes ^2^H and ^18^O, were injected into the animals (60% ^18^O and 30% ^2^H; approximately 0.5 ml, exact volume known by weighing the syringe before and after injection), and CO_2_ production was calculated based on the differential washout of the ^2^H and ^18^O isotopes over a period of 24 h – 72 h. We used the two-sample method [25], taking an initial blood sample about one hour after injecting the isotopes (after equilibration with the body water pool) and immediately after recapture [25]. Capillaries that contained blood samples were vacuum distilled [24], and water from the resulting distillate was used to produce CO_2_ and H_2_ (methods in [25] for CO_2_, and [5] for H_2_). The isotope ratios ^18^O:^16^O and ^2^H:^1^H were analysed using gas source mass spectrometry (Optima, Micromass IRMS and Isochrom μG, Manchester, UK) and were converted to DEE using a single pool model as recommended for animals of this size [25] and used previously for this species [26]. CO_2_ production was converted to DEE assuming a respiratory quotient of 0.85. In the calculations we assumed a fixed evaporation of 25% of the water flux (equation 7.17 [25]). Eighty-nine percent of final blood samples were obtained within three hours of a 24 h interval from the initial blood sample, which controlled for circadian rhythms of activity (max  =  7.2 h from 24 h interval [27], [28]). The average body mass of weasels, based on values obtained at the beginning and end of the DLW technique, was used in all analyses. In analyses where DEE was the dependent variable, we averaged all T_a_s taken at 08:00, 14:00, and 20:00 over the DLW interval.

### Resting metabolic rate

We measured the RMR of male weasels 89 times on 54 different weasels, in 2004 (n = 19), 2005 (n = 39), 2006 (n = 28) and 2007 (n = 3). Twenty-four RMR measures were paired with DEE measures on the same individuals. For all individuals, we attempted to obtain RMR measures immediately prior to the DLW injection. In the instances where we failed to obtain a RMR measure prior to the DLW injection (e.g. the individual did not rest in the chamber), we performed the RMR measure following the final blood sample of the DLW technique. As a result, the paired RMR estimates were determined within four days on either side of the final blood sample of the DLW technique. We also made 65 additional estimates of RMR that were not paired with DEE estimates.

We used a positive-pressure, open-circuit respirometry system to quantify RMR. Dry atmospheric air (Drierite, Hammond Drierite Co. Xenia, OH, USA) was pushed through a copper coil submerged along with the metabolic chamber in a water bath to control the temperature within the thermal neutral zone of weasels (30°C [29]). Prior to the metabolic chamber, the airstream was divided into control and measurement streams which were controlled by separate mass flow controllers (Sierra Instruments, Monterey, CA, USA or ERG-1000, Warsaw, Poland). The measurement stream was pushed through a metabolic chamber (2300 cm^3^) at a mean rate of 900 mL/min. The measurement gas stream was then re-dried (Drierite), sub-sampled at a rate of 200 mL/min, and then passed through a FC-10b oxygen analyzer (Sable Systems, Las Vegas NV, USA). Digital signals from the analyzer were stored using WinWedge 3.0 (Taltech, Philadelphia, PA, USA) and subsequently analyzed with DATACAN V (Sable Systems). We calculated oxygen consumption rates using equation five in [30] and presented RMR in kJ/day assuming RQ  =  0.8 (i.e. 20.1 kJ/L O_2_, [31]). All metabolic trials were carried out at night, starting at 20:00. Before each trial, weasels were fed only in the morning, so they were not fasted longer than 12 hours before the RMR measurement. In the analyses, we used the body mass taken directly before the RMR trial.

### Statistical analyses

Inspection of the residuals from all models suggested that they were normally distributed. We used linear mixed-effects models (R library: lme4) to examine the predictors of AT, DEE, and RMR. These models included the random factors year and weasel ID. The fixed effects were body mass, T_a,_ and habitat (meadows and marshes). Body mass was included as a covariate in the analyses of AT, DEE, and RMR. The significance of fixed effects was assessed using stepwise backwards elimination. The fixed effects with the largest *P* values were removed first, retaining all effects with *P* ≤ 0.05. The significance of the fixed and random effects was determined using likelihood ratio tests (–2 times the difference in log-likelihoods between hierarchical models estimated using maximum likelihood, tested against a χ2 distribution with the number of degrees of freedom equal to the difference in the number of terms estimated).

We used quantile regression (R library: quantreg, [32]) analyses to estimate the relationship between T_a_ and maximum values of AT, DEE, RMR and metabolic scope. Quantile regression is particularly useful for defining limiting effects of covariates by constructing models for upper quantiles of the conditional distribution [32], by fitting data with linear functions for different parts of the response variable distribution. The significance and standard errors of the coefficients were calculated by bootstrapping.

All analyses were performed using R [33] and means and model coefficients are presented ± standard error.

## Results

**Figure 1:**
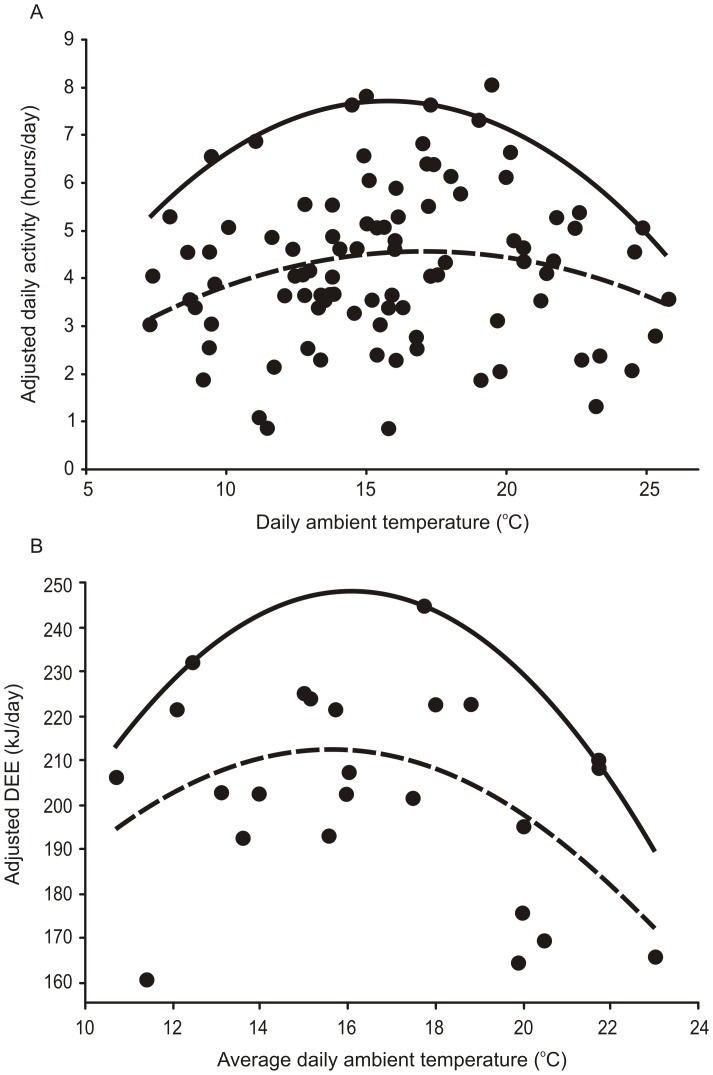
Effect of ambient temperature on activity time and daily energy expenditures. The quadratic effect of average daily T_a_ (ambient temperature) on (A) AT (daily activity time; hours/day) adjusted for the effect of habitat; (B) DEE (daily energy expenditure; kJ/day) adjusted for the effects of body mass and AT, in male weasels. Broken line – fitted using parameters from mixed model (mean response), solid line - fitted by linear quantile regression (for *tau*  =  0.95).

**Figure 2:**
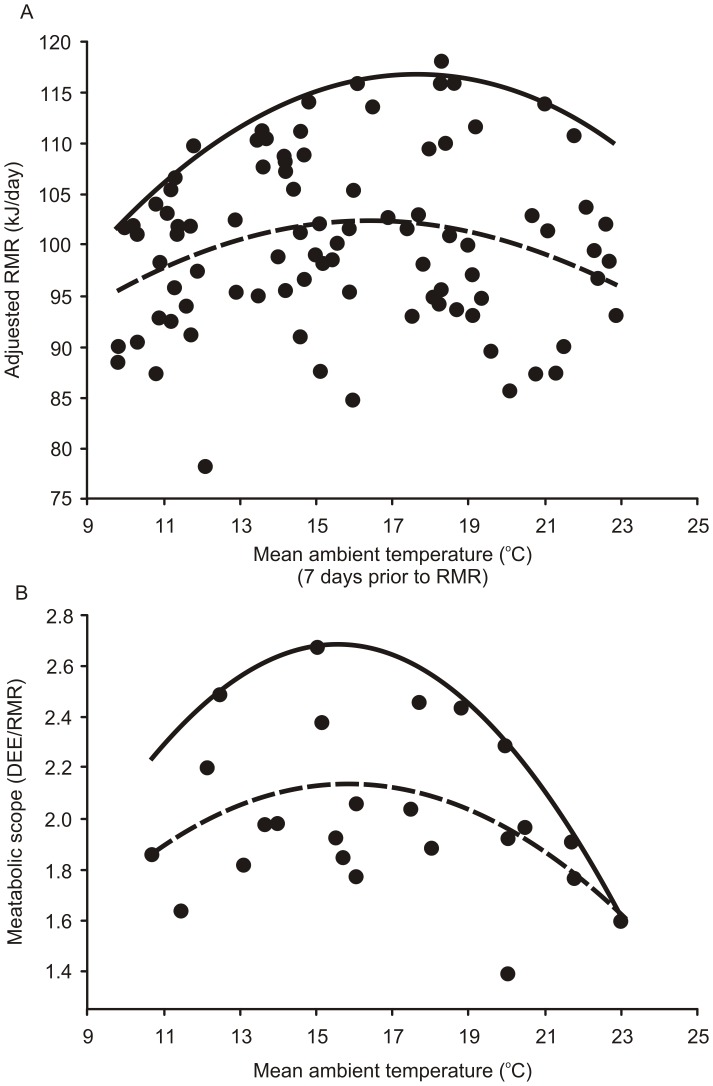
Effect of ambient temperature on resting metabolic rate and metabolic scope. The quadratic effect of mean T_a_ (ambient temperature) on (A) RMR (resting metabolic rate; kJ/day) adjusted for the effects of body mass, and (B) metabolic scope (DEE/RMR), in male weasels. Broken line – fitted by linear least-square regression (mean response), solid line - fitted by linear quantile regression (for *tau*  =  0.95).

Weasels were active for 4.2±0.2 hours/day. AT showed a “hump-shaped” (i.e. convex) quadratic response to T_a_ (Table 1, Fig. 1A). Based on the line of best fit, AT, adjusted for the other significant fixed effects in Table 1, peaked at 17.0°C and was 31% and 25% less at the minimum and maximum daily T_a_s at which AT was examined, respectively (Table 1, Fig. 2A). Body mass was not a significant predictor of daily AT (*P* = 0.82). The random effect of ID was significant in the final model (*P* = 0.01), but the random effect of year was not significant (*P* = 0.92). The quantile regression analysis revealed a significant hump-shaped relationship between T_a_ and maximum AT (for *tau* = 0.95 both linear and squared terms were significant at *P* < 0.01, Fig. 1A).

**Table 1:** Estimates of model coefficients (± standard error) for significant predictors of AT (daily activity time; hours/day) and DEE (daily energy expenditure; kJ/day) in male weasels, derived from a linear mixed-effects model (random effects were weasel ID and year).

Predictor	Coefficient ± SE	χ^2^	*P*
AT - daily activity time			
T_a_	0.64±0.31	6.93	0.03
T_a_^2^	–1.75^e−2^±7.04^e−3^	5.96	0.01
Habitat	–0.96±0.42	4.79	0.03
DEE - daily energy expenditure			
Body mass	1.96±0.18	42.60	< 0.0001
T_a_	50.81±14.85	9.89	0.007
T_a_^2^	–1.28±0.37	10.00	0.002
AT	10.28±2.94	11.80	0.003
T_a_ : AT	–0.56±0.19	7.25	0.007

T_a_ – mean daily ambient temperature (°C).

The average DEE of weasels was 203.6±8.2 kJ/day (average mass  =  96.2±3.6 g). Similar to AT, DEE also showed a hump-shaped quadratic response to T_a_ (Table 1, Fig. 1B). Based on the line of best fit, DEE, adjusted for the other significant effects in Table 1, peaked at 16.0°C and was 8% and 19% less at the minimum and maximum daily temperatures at which DEE was examined, respectively (Table 1, Fig. 1B). Body mass was positively related to DEE (Table 1, Fig. 2B). Both the random effects of ID and year were nonsignificant (*P* = 0.96). AT was positively correlated with DEE and the interaction between AT and T_a_ was also significant (Table 1). In the quantile regression analysis, maximum DEE showed a hump-shaped relationship with T_a_ (quantile regression, for *tau* = 0.95, *P* = 0.04 and *P* = 0.03, for linear and squared term of T_a_, respectively, Fig. 1B). AT was not correlated with maximum DEE (quantile regression, for *tau* = 0.95, *P* = 0.49).

In the full dataset, RMR averaged 100.3±12.1 kJ/day (average mass  =  96.3±15.9 g). RMR was significantly correlated with body mass (coefficient  =  1.24±0.12, t = 10.02, P < 0.0001), and with linear (coefficient  =  11.38±3.98, t = 2.86, P = 0.005) and quadratic (coefficient  =  –0.35±1.12, t = –2.80, P = 0.007) effects of T_a_ calculated by averaging the daily mean T_a_ values from the seven days prior to the RMR measurement (Fig 2A). A time span of seven days was selected by applying different time windows (from 1 to 21 days) and choosing the model with the greatest R^2^ value. Maximum RMR was related to T_a_ in a hump-shaped manner (quantile regression, *tau* = 0.95, P < 0.001 for linear and squared terms, Fig. 2A).

Metabolic scope varied between 1.39 an 2.67. Similar to AT, DEE, and RMR, metabolic scope also showed a hump-shaped quadratic response to T_a_ (Table 2, Fig. 2B). Neither individual ID, nor year were significant random factors in the model. The maximum values of metabolic scope showed a hump-shaped response to T_a_ (quantile regression, for *tau* = 0.95, P = 0.002 and P = 0.001, for linear and squared terms of T_a_ respectively, Fig. 2B).

**Table 2:** Estimates of model coefficients (± standard error) for the effect of body mass, mean ambient temperature (T_a_) and habitat type on metabolic scope (DEE/RMR) in male weasels.

Predictor	Coefficient ± SE	χ^2^	*P*
Body mass	0.01±0.002	17.23	< 0.001
Habitat	0.28±0.09	8.40	0.004
T_a_	0.27±0.11	5.58	0.02
T_a_^2^	–0.01±0.003	6.58	0.01

## Discussion

In our previous paper [26] we demonstrated that the maximum DEE of male weasels was relatively constant across a temperature range of –20°C to 20°C, mainly due to high thermoregulatory costs in winter. We also provided evidence that male weasels are able to avoid high DEEs by reducing their activity time in response to cold T_a_s. While our previous work focused on the influence of cold T_a_s on the AT and DEE of male weasels, the present work addresses the effect of high temperatures on the AT and DEE of male weasels during the breeding season. Our results show that weasels are less active on days with warmer T_a_s. DEE showed a similar hump-shaped response to T_a_, presumably because lower AT directly reduced the energetic costs associated with activity and indirectly reduced energetic costs associated with thermoregulation. Inactive weasels spend the majority of their time in the underground nests and shelters of rodents that they take over after a kill [23], where conditions are close to thermoneutral [34], [35]. In our analyses we detected a significant AT by T_a_ interaction. This interaction suggests that the positive effect of AT on DEE was stronger at colder T_a_s than at warmer T_a_s. The most likely interpretation of this interaction is that thermoregulatory energetic costs associated with being active are greater at colder T_a_s than at warmer T_a_s.

One possible explanation for the link between T_a_ and AT that we can rule out is that the observed pattern reflects the activity of microtine rodents, the primary prey of weasels in our study area [21]. The activity rhythm of these rodents are characterized by several short-term activity cycles without any regular pattern [36]. Thus, regardless of whether rodents are easier for weasels to hunt when they are active [37], (but see [21], [38], [39]), or when they are in their nests [25], the hunting periods of weasels should be independent of T_a_.

Previous explicit tests of the HDL hypothesis have occurred on laboratory animals exposed to a limited number of T_a_s (typically two or three T_a_s; e.g. [40]–[42]). As a result, the ability of these studies to resolve the shape the relationship between T_a_ and metabolic performance is limited. Weasels in our study that were exposed to natural variation in T_a_ showed a hump-shaped relationship between T_a_ and both AT and DEE. Thus, our results suggest that heat dissipation only plays a role in shaping AT and DEE above the temperatures where these two measures peaked (17°C for AT and 16°C for DEE). Below these temperatures, the AT and DEE of weasels is clearly shaped by other factors other than the capacity to dissipate heat. Future work is clearly required to determine how the factors influencing AT and DEE change with respect to T_a_.

The decline of AT and DEE on days with high T_a_s is consistent with the HDL hypothesis. This hypothesis suggests that AT and DEE was reduced at high T_a_s because of negative physiological effects associated with hyperthermia. The average daily T_a_s above which we found that AT and DEE started decrease were 17°C and 16°C, respectively. Past research suggests that the lower critical temperature of the thermalneutral zone for weasels at rest is between 25°C and 30°C in the summer [29]. However, there are no data on the T_a_ above which active weasels face the risk of hyperthermia. Weasels have higher than predicted costs of locomotion based on their size [43] and presumably on warm days individuals face the risk of hyperthermia while active as a result of the heat generated from their costly mode of locomotion.

Our results suggest that T_a_ influenced DEE by influencing AT. When AT decreased (at very low and high T_a_s) thermoregulatory demands have limited effect on DEE because inactive weasels stay in thermoneutral conditions in rodents nests. However, costs of thermoregulation become a substantial part of DEE when AT increases at low temperatures (see also [26]), whereas heat dissipation becomes limiting factor at high end of temperature range.

Our observation that weasels during the breeding season had lower RMRs when the previous seven days were warm is also consistent with predictions of the HDL hypothesis [4]. In weasels, the energy expended while individuals are inactive, and thus presumably at RMR levels [34], [35], comprises roughly 50% of their DEE [22]. Although research on free-ranging animals is critically lacking, reviews of the literature based on laboratory studies suggest that the energy demands of small animals at rest comprises 35% of DEE [44]. This percentage in weasels is likely high as compared to other small mammals because they spend most of their day inactive (24 hours – 4.2 hours active [see Results]  =  19.8 hours) and the RMR of weasels is roughly twice as high as in other animals of similar size [29]. The high proportion of the energy budget of weasels that is spent at rest makes it possible that the plastic reductions in RMR that we see in response to warm temperatures can lead to reductions in daily energy demands, and presumably the amount of time that weasels need to be active to meet these demands. Past research has demonstrated that herbivorous desert mammals can reduce their RMR when exposed to short-term starvation [45]–[47]; however, this is the first case of a free-living mammal reducing its RMR in a temperate environment. Our finding that weasels plastically reduce their RMR in response to warm T_a_s raises the question of why weasels would ever maintain a high RMR when they can survive with a lower RMR. A possible explanation is that there are fitness costs associated with plastic reductions in RMR. However, considerable research is required to determine whether a reduced RMR compromises survival (e.g. via reduced immune function, [48]) or reproductive success (e.g. via reduced aerobic performance and mate searching, [16], [17]).

A negative effect of T_a_ on AT has also been observed in other predators that have energetically demanding lifestyles [19], [49]–[54]. Wolves significantly reduced their movement on days when mean daily temperatures exceeded 20°C [39]. Theuerkauf et al. [39] analyzed 11 studies on wolves and found a positive relationship between T_a_ and the nocturnal index, which reflects the extent to which animals were active during the night relative to during the day [55]. Hayward and Hayward [56] found that adult male lions, characterized by darker pelage that absorbed more solar radiation, and young spotted hyenas *Crocuta crocuta* with longer fur, restricted their activity to cooler night-time periods. Moreover, in Iberian lynx (*Lynx pardinus)* and Canadian lynx (*Lynx canadensis*), the amount of time spent active were negatively correlated with summer temperatures [38], [57]. Finally, badgers (*Meles meles*), which are primarily active at night, are less active at temperatures above 17°C [58].

The avoidance of overheating is not limited to actively hunting predators. Female mountain goats *Oreamnos americanus*, a species adapted to cold temperatures most of the year, spend less time foraging overall and forage more at dusk during warm days [59]. Moreover, lactating red squirrels occupy less insulated nests when facing greater heat loads [60]. Finally, migrating birds often fly at high altitudes and during the night, which may function to dissipate the body heat generated during prolonged flight [61].

The results of this study and the existing data therefore provide many examples that heat avoidance is a widespread phenomenon, which was previously overlooked or misinterpreted. Although the activity and energy expenditure of large endotherms are most likely to be constrained because they are less able to dissipate heat [4], our results suggest that small endotherms may also experience constraints consistent with the HDL hypothesis. The significance of the HDL theory in explaining ecological patterns and processes may increase as climate change progresses. There are well established predictions how increasing mean temperatures will affect the distribution of many taxa, e.g. [62], [63], and the ability to dissipate heat may be one of the crucial factors limiting performance.
